# External Genitalia Myiasis in a 40-Year-Old Woman

**DOI:** 10.1155/2023/5579531

**Published:** 2023-08-09

**Authors:** Ghazal Mansouri, Leila Allahqoli, Hamid Salehiniya, Ibrahim Alkatout

**Affiliations:** ^1^Department of Obstetrics and Gynecology, School of Medicine, Afzalipour Hospital, Kerman University of Medical Sciences, Kerman, Iran; ^2^Ministry of Health and Medical Education, Tehran, Iran; ^3^Social Determinants of Health Research Center, Birjand University of Medical Sciences, Birjand, Iran; ^4^Department of Obstetrics and Gynecology, University Hospitals Schleswig-Holstein, Campus Kiel, Kiel, Germany

## Abstract

Human myiasis is an infestation produced by fly larvae invading the tissues. We present a case of a 40-year-old virgin woman with vulvar myiasis. She reported at the gynecology clinic with a bloody discharge, severe pain, and swelling of the genital area for six days. Her menstrual history revealed the use of folded clothes. She had no specific gynecological disease. At the examination of the external genitalia, a tender mass measuring 6 cm × 4 cm and an ulcer measuring 1 cm × 1 cm on the surface of the labia majora were found. The patient was hospitalized. Serology, blood, and urine tests were requested; all laboratory tests were normal. The patient was transferred to the operating room (OR) with the diagnosis of necrotizing fasciitis. In the OR, we performed a longitudinal incision on the mass and removed nearly 30 visible maggots. After washing with normal saline, the patient was transferred to the ward without wound suturing. Debridement of the necrotic vulvar mass along with daily washing was performed for 7 days. The wound was sutured on the seventh day at the OR. Antibiotic therapy was continued for 4 days, and the patient was discharged with normal laboratory tests on the eleventh day after admission. We believe that poor sanitary hygiene was the cause of vulvar myiasis in our patient. We conclude that appropriate measures must be taken to reduce the risk of human myiasis, especially in tropical rural regions.

## 1. Introduction

Myiasis is a parasitic infection of living or dead tissues of a host by the larvae of various species of flies [1]. The larvae of four flies, *Cochliomyia hominivorax*, *Sarcophaga haemorrhoidalis*, *Dermatobia hominis*, and *Lucilia eximia*, were identified as the causative agents in reported clinical cases [2]. Larvae, such as *D. hominis*, *Cordylobia rodhaini*, or *Chrysomya bezziana*, usually require the ingestion of living tissue to complete their life cycles, thus causing additional tissue damage [3, 4]. Although the entry of the larvae occurs through skin wounds or body cavities, such as the eyes, ears, mouth, or genitourinary tract [1], they may penetrate the skin directly [5]. Myiasis in humans occurs in warm, humid tropical and subtropical regions [6]. This type of infestation in humans is linked with poor socio-economic conditions and favorable climatic conditions for flies [7]. Myiasis is usually observed in young neglected children, very old patients, mentally retarded, and bedridden patients who are unable to take care of themselves [8]. Patients with severe burn injuries and poorly managed wounds may also be infested by fly larvae [4]. Infestation in young, healthy, mentally sound, and active persons is rare. Covered body parts, such as the genitals, are a rare location for the infestation. Myiasis of the vulva constitutes a mere 0.7% of all cases of human myiasis [8]. We report, by the SCARE criteria, a case of a 40-year-old woman with vulvar myiasis [9].

## 2. Case Report

A 40-year-old woman from a poor socioeconomic background, residing in a rural area of southern Iran (Kerman), was admitted on 22 October 2021 with a bloody discharge, severe pain, and swelling of the genital area. The pain and swelling had been started six days before admission. She had sought medical help for an ulcer and bloody discharge at Jiroft Hospital and was hospitalized for three days. She received antibiotics and was then referred to our tertiary University center because her condition did not improve. She had no gynecological disease. Her menstrual cycle was regular, and she used folded cloths instead of sanitary pads during her menstruation. Moreover, she washed the clothes with water and hung them on the clothesline to dry before reuse. Her medical history revealed no evidence of any underlying disease (immunosuppression, chronic illness, such as diabetes or tuberculosis, and steroid therapy) or mental disorder. She had no history of trauma or insect bite. Her physical examination was normal, although she had been blind since childhood.

The patient had no abdominal pain, gastrointestinal or urinary symptoms, fever, or chills. She did have a history of COVID-19 infection in June 2021, from which she recovered spontaneously without being hospitalized. At admission, her vital signs were as follows: pulse rate 86 bpm; temperature 37.2°C; BP 110/70 mmHg; and O_2_ 97%. We found no evidence of lymphadenopathy. Auscultation of her heart and lungs revealed normal conditions. The examination of her abdomen disclosed no tenderness or rebound tenderness.

Before examination of her external genitalia, she reminded us that she was a virgin, and had not had sexual intercourse throughout her life. Serology, blood, and urine tests were requested.

**Figure 1:**
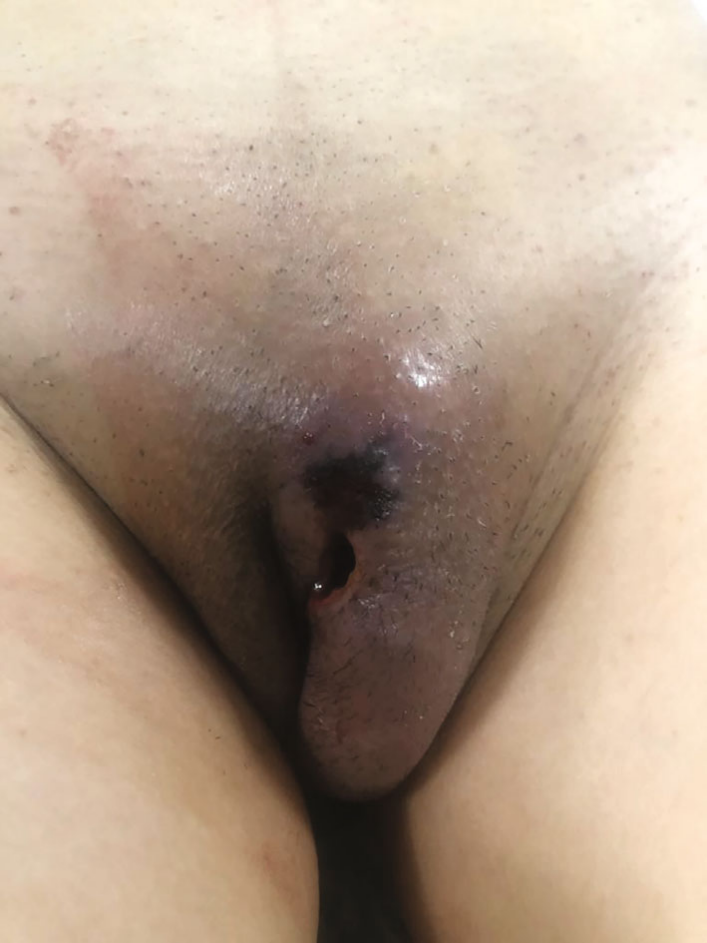
External view of the genitalia.

The examination of the external genitalia revealed a tender mass measuring 6 cm × 4 cm in the vulvar area and an ulcer measuring 1 cm × 1 cm next to the clitoris (Figure 1). In the clitoral region, the patient had a bloody discharge.

**Figure 2:**
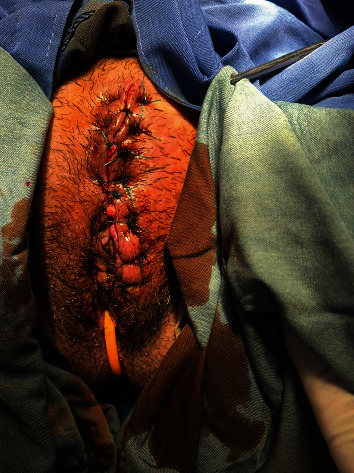
External view of the genitalia after healing of the wound.

The patient's total and differential leukocyte counts were within normal limits, and her hemoglobin level was 14.2 g/dL. The results of a complete urine examination revealed normal values. Serological tests for the human immunodeficiency virus and syphilis (rapid plasma reagin card test) were negative. Counseling in regard to infectious disease and surgery counseling was requested. The patient was then transferred to the operating room (OR) with the assumed diagnosis of necrotizing fasciitis. A longitudinal incision was performed on the mass under sterile conditions. Necrotic fluid was drained, and approximately 30 visible maggots were removed with non-toothed forceps. Necrotic tissue, which was largely superficial and subcutaneous, was debrided completely. The fascia and muscles were not involved. After washing with normal saline, the patient was transferred to the ward without wound suturing. Debridement of the necrotic vulvar mass along with daily washing was performed for 7 days. Some maggots were seen until the third day and removed. This was followed by surgical lavage. Intravenous clindamycin (900 mg three times a day) and ceftriaxone (1 g twice daily) were started to prevent secondary bacterial infection. The wound was sutured on the seventh day (Figure 2). Antibiotic therapy was continued for four days, and the patient was discharged with normal lab tests on the eleventh day after admission. The wound culture-confirmed larvae of the blowfly. At the time of discharge, the patient was advised to keep the vagina and vulva clean.

## 3. Discussion

Myiasis is an infestation of the skin by developing larvae. Multiple species of flies are indigenous to various parts of the world [10, 11]. Some fly lay their eggs on or near a wound or sore, and the larvae burrow into the skin when they hatch [12]. Depending on the respective conditions, it takes approximately eight hours to a day for the eggs to hatch. The larvae lacerate the skin with their mouths once they have hatched, causing open sores. The larvae burrow through the wounds into the host's subcutaneous tissue once the skin has been pierced, generating deep, and itchy lesions that are extremely susceptible to infection. Bacterial infection probably occurs after the second day and, if left untreated, may cause bacterial infection or sepsis in the bloodstream. If left untreated, the affliction may cause anorexia and frailty and is usually fatal [13].

Genital myiasis is a rare condition; a small number of cases have been reported to date [14]. Our case is an interesting and rare example of vulvar myiasis; maggots had invaded the labia majora. The flies may have laid eggs on the undergarment or fabric that the patient was wearing during her menstrual period, as they were usually hung outside after being washed. The eggs might have been transported to the vulva via a soiled undergarment or fabric. Transmission of fly eggs to the genitals through dusty clothing is the most commonly accepted mechanism of genital myiasis [15].

Urogenital myiasis has been largely reported in Asia and South America between 1975 and 2017, primarily in India and Brazil [12]. Poor sanitation, a low socioeconomic status, poor mental or physical health, poor self-hygiene, cervical cancer, the presence of a urethral stent, sexually transmitted diseases (STDs) [16, 17], lack of mobility, old age, and ulcerated and prolapsed organs are all risk factors for genital myiasis [18].

Since myiasis is a rare condition with no identifiable symptoms, physicians are generally not trained to recognize it [19]. Myiasis is frequently misdiagnosed [20], resulting in unnecessary therapy [21]. The gynecologist must be able to distinguish genital myiasis from other chronic granulomatous infections of the vulva, STDs, and malignancies [22]. The diagnosis relies on a thorough understanding of the clinical entity and a detailed examination of the lesions.

## 4. Conclusion

Vulvar myiasis is a very rare occurrence. Awareness of its characteristic clinical features and careful inspection of the lesions are key aspects of the diagnosis. Pain, swelling, and redness are easily confused with STDs. Despite its rarity, vulvar myiasis is a preventable and treatable disease. Its prevention calls for concentrated efforts to increase awareness of genital hygiene in non-affluent countries.

## Data Availability

All data generated or analyzed during this study are included in this published article.
